# An analytical approach to sparse telemetry data

**DOI:** 10.1371/journal.pone.0188660

**Published:** 2017-11-28

**Authors:** Michael J. Kinney, David Kacev, Suzanne Kohin, Tomoharu Eguchi

**Affiliations:** 1 Ocean Associates; Under Contract to Southwest Fisheries Science Center, National Marine Fisheries Service, National Oceanic and Atmospheric Administration, San Diego, California, United States of America; 2 Southwest Fisheries Science Center, National Marine Fisheries Service, National Oceanic and Atmospheric Administration, San Diego, California, United States of America; Public Library of Science, FRANCE

## Abstract

Horizontal behavior of highly migratory marine species is difficult to decipher because animals are wide-ranging, spend minimal time at the ocean surface, and utilize remote habitats. Satellite telemetry enables researchers to track individual movements, but population level inferences are rare due to data limitations that result from difficulty of capture and sporadic tag reporting. We introduce a Bayesian modeling framework to address population level questions with satellite telemetry data when data are sparse. We also outline an approach for identifying informative variables for use within the model. We tested our modeling approach using a large telemetry dataset for Shortfin Makos (*Isurus oxyrinchus*), which allowed us to assess the effects of various degrees of data paucity. First, a permuted Random Forest analysis is implemented to determine which variables are most informative. Next, a generalized additive mixed model is used to help define the relationship of each remaining variable with the response variable. Using jags and rjags for the analysis of Bayesian hierarchical models using Markov Chain Monte Carlo simulation, we then developed a movement model to generate parameter estimates for each of the variables of interest. By randomly reducing the tagging dataset by 25, 50, 75, and 90 percent and recalculating the parameter estimates, we demonstrate that the proposed Bayesian approach can be applied in data-limited situations. We also demonstrate how two commonly used linear mixed models with maximum likelihood estimation (MLE) can be similarly applied. Additionally, we simulate data from known parameter values to test each model’s ability to recapture those values. Despite performing similarly, we advocate using the Bayesian over the MLE approach due to the ability for later studies to easily utilize results of past study to inform working models, and the ability to use prior knowledge via informed priors in systems where such information is available.

## Introduction

Where and why animals move remain relatively poorly understood population processes. Underlying these simple questions are complex interactions among life history, physiology, behavior, forage distributions, and habitat, which make understanding movement a difficult endeavor. A myriad of techniques have been developed to study the movement of animals across terrestrial, marine, and aerial environments. Some are direct methods such as visual observations, or tagging [1–6]; while others are indirect such as genetics or stable isotopes [7–11].

Our focus in this study is on direct methods, specifically those using electronic tagging technology. Over the last several decades the scientific literature has seen a steady expansion in the number of studies utilizing various forms of electronic tags to record data on animal movement and associated environmental parameters [12, 13]. For various reasons (concealment, remoteness, etc.) observations of marine animal movement have proven particularly challenging, most notably for large pelagic fish species like sharks, tuna, and billfish, which can cover vast distances and do not require regular trips to the surface to breathe [e.g. 14, 15–17]. For these animals such challenges have resulted in various levels of data paucity. Many of the currently available meta-analytical statistical methods require data to meet certain basic criteria, such as having one data point per day of a track (or the ability to interpolate to that level), or information on movement speed and or turning angle to accurately define a behavioral state [e.g. 18, 19, 20]. In data-limited situations, these prerequisites restrict available analytical approaches. While not true of all studies, these data restrictions lead many researchers to approach their data at the individual level in a qualitative, largely descriptive manner, rather than quantitatively and at a population level as indicated by Jonsen et al. [21], Heupel et al. [22], and Papastamatiou and Lowe [23].

Restricting these data limited tagging studies to qualitative analysis at the individual level can lead to gaps in our knowledge of population level movements, often resulting in established paradigms being repeatedly stated by successive studies. Common Thresher sharks (*Alopias vulpinus*) and Lemon sharks (*Negaprion brevirostris*) are examples of species that have seen extensive amounts of tagging work with most to all of the analysis focused at the individual level [24–27]. In the case of Threshers this has led to hypotheses about population level seasonal migrations established in the late 1980s and early 1990s based on CPUE of the California drift gill net fishery to persist in the literature and be used as the basis for seasonal movement in stock assessment work [28]. Despite advances in statistical techniques, without a clear approach for data limited situations, the owners of such data will likely continue to couch their analyses in more qualitative methods and stick to established paradigms, meaning these kinds of situations are likely to persist.

Here we seek to spur the use of quantitative methods to investigate population level questions with telemetry data, even when data are sparse. Questions such as the likelihood of movement in and out of marine protected areas, or across state and international boundaries, ontogenetic shifts in habitat use, and the impacts of various environmental conditions on such movements. We outline three approaches to analyzing telemetry data and provide a guide for using them, as well as to test their robustness to various levels of data paucity. In keeping with our focus on data limited situations our approaches do not require regular data intervals or measurements of speed, and turning angles. They do however, require that variables of interest and the modeling question itself be scaled properly relative to the uncertainty in available data, and that questions be designed with a binary response variable (more on this below).

The approaches presented here in and of themselves are not novel; neither is putting them in sequence to provide a robust framework for testing model assumptions. Instead, these steps are laid out and tested for their performance under various levels of data paucity, to provide a framework that can be applied in data limited situations. To test the effects of data paucity on population level inferences, we had to find a robust dataset and randomly pare it down to assess if the smaller datasets provided the same inferences as the full set. We also use simulated data, which are described in greater detail below, to assess each approach’s robustness to data availability. Parameter estimates from increasingly sparse datasets are directly compared, allowing us to discuss the effects of limited data on inferences. We endeavor with this work to make these statistical tools available to a broad and perhaps less quantitative audience, as well as to spur the use of quantitative methods in data limited situations.

## Methods

### General overview

Three modeling approaches are presented here: a Bayesian approach using Just Another Gibbs Sampler (JAGS) (R package: rjags [29]), and two generalized linear mixed models (GLMM), penalized quasi-likelihood (PQL) (R package: MASS [30]), and “glmer” using the Laplace approximation (R package: lme4 [31]). Given our emphasis on data limited situations our approaches focus on binary questions e.g., whether an animal migrates across the equator or from one known habitat to another, or when an animal switches from one behavior to another, such as from foraging to transiting (e.g., [32]). Given telemetry data, and a binary response variable, these approaches attempt to estimate parameter values that relate variables of interest such as environmental conditions (various environmental indices, sea surface temperature, etc.) or demographic values (sex, size, etc.) to that response variable. All analyses presented here are conducted in R (version 3.2.3; [33]).

As discussed by Bolker et al. [34], describing the use of GLMMs or Bayesian models to analyze data necessarily touches on controversial statistical issues such as the debate over null hypothesis testing, or the use of informative or vague priors. These topics have been thoroughly covered by others [35–37] and further discussion is outside the scope of this paper. These discussions aside, however, comparing the results of GLMMs and Bayesian models in various states of data paucity seems prudent when providing a practical framework in the hopes of spurring a more quantitative approach to sparse telemetry data.

Throughout this paper we deal with two datasets. The first is a real satellite-linked radio-telemetry-tag (SLRT) dataset of Shortfin Mako (*Isurus oxyrinchus*; hereafter Mako) in the northeast Pacific provided by the Southwest Fisheries Science Center. This dataset was selected because of the abundance of data (9440 locations across 34 tracked individuals), which allowed us to randomly pare it down to create “data poor” subsets to test the effects of data paucity on population level inferences. The second dataset, a simulated Mako dataset generated to mirror the real Mako dataset in size and level of individual variation, but with known, true parameter values. Both datasets were subjected to various levels of subsetting to assess the effects on inferences. The simulated dataset alone would be adequate for testing the effects of limited data on model inferences however; our desire to provide a framework for a broad audience will be assisted by showing our model outcomes when using both a real world and a simulated examples.

**Fig 1:**
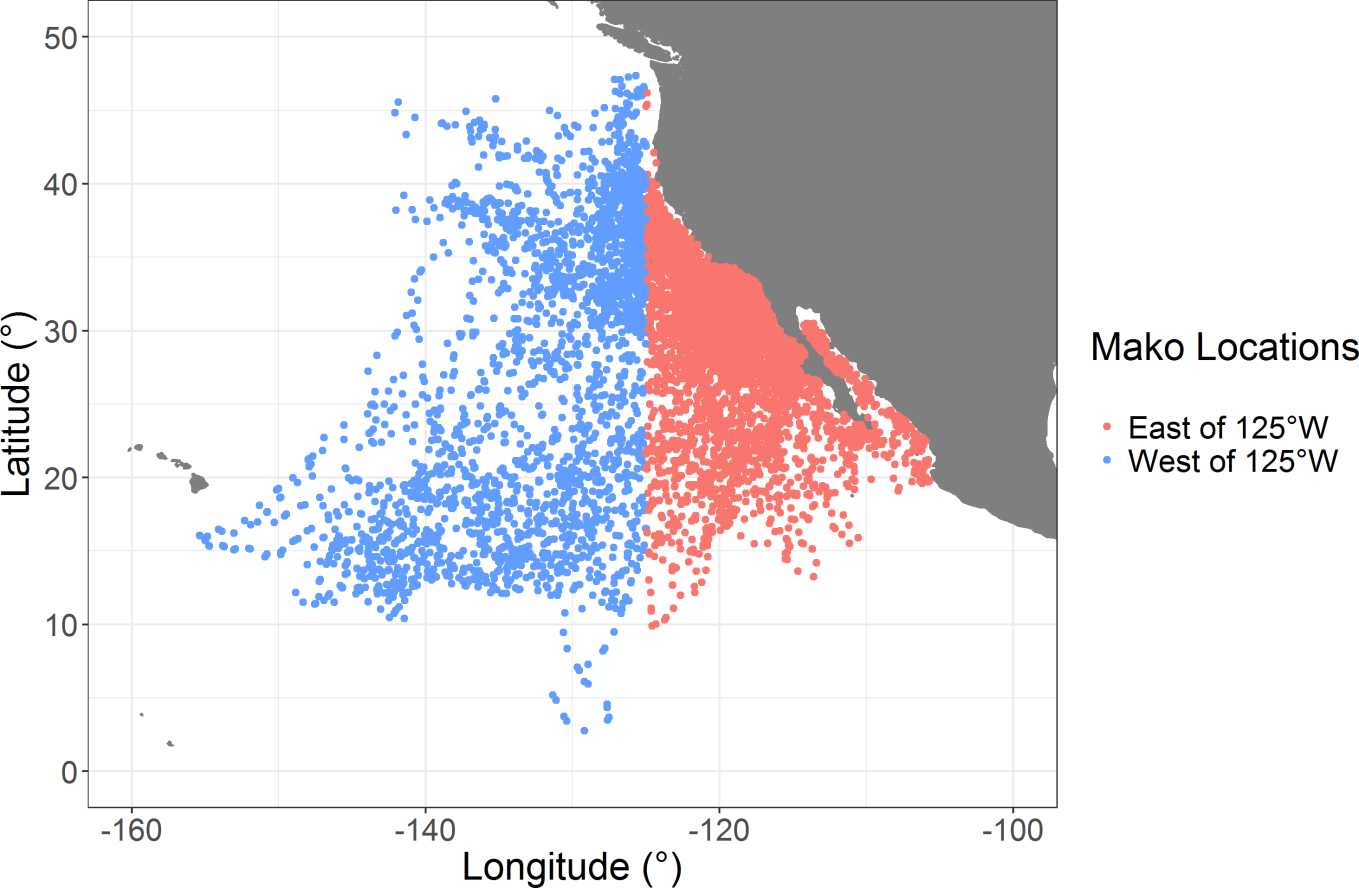
Map of Mako locations. Aggregated SLRT locations for 34 individual Makos (9440 total locations). Points in red are east of 125°W longitude boundary. Points in blue are in west of the boundary.

With the focus here being on methods, a meaningful discussion of Mako ecology is beyond the scope of this work. As such, our binary question concerns whether Mako were found east or west of an arbitrary line (longitude 125˚W) (Fig 1). This question was not selected for its ecological relevance, but more to provide an interpretable example of a binary question about horizontal movement. With this question, we outline a practical framework for the use of quantitative tools by stepping through each stage of analysis while providing sample data and R code. We first describe the preparation of data (i.e. refining locations and standardizing variables to comparable scales). We then develop a linear model through the process of variable selection using Random Forest, and test to confirm variable linearity using a Generalized Additive Mixed Model (GAMM). Finally, we develop both Bayesian and GLMM frameworks to analyze the data.

### Data acquisition

**Fig 2:**
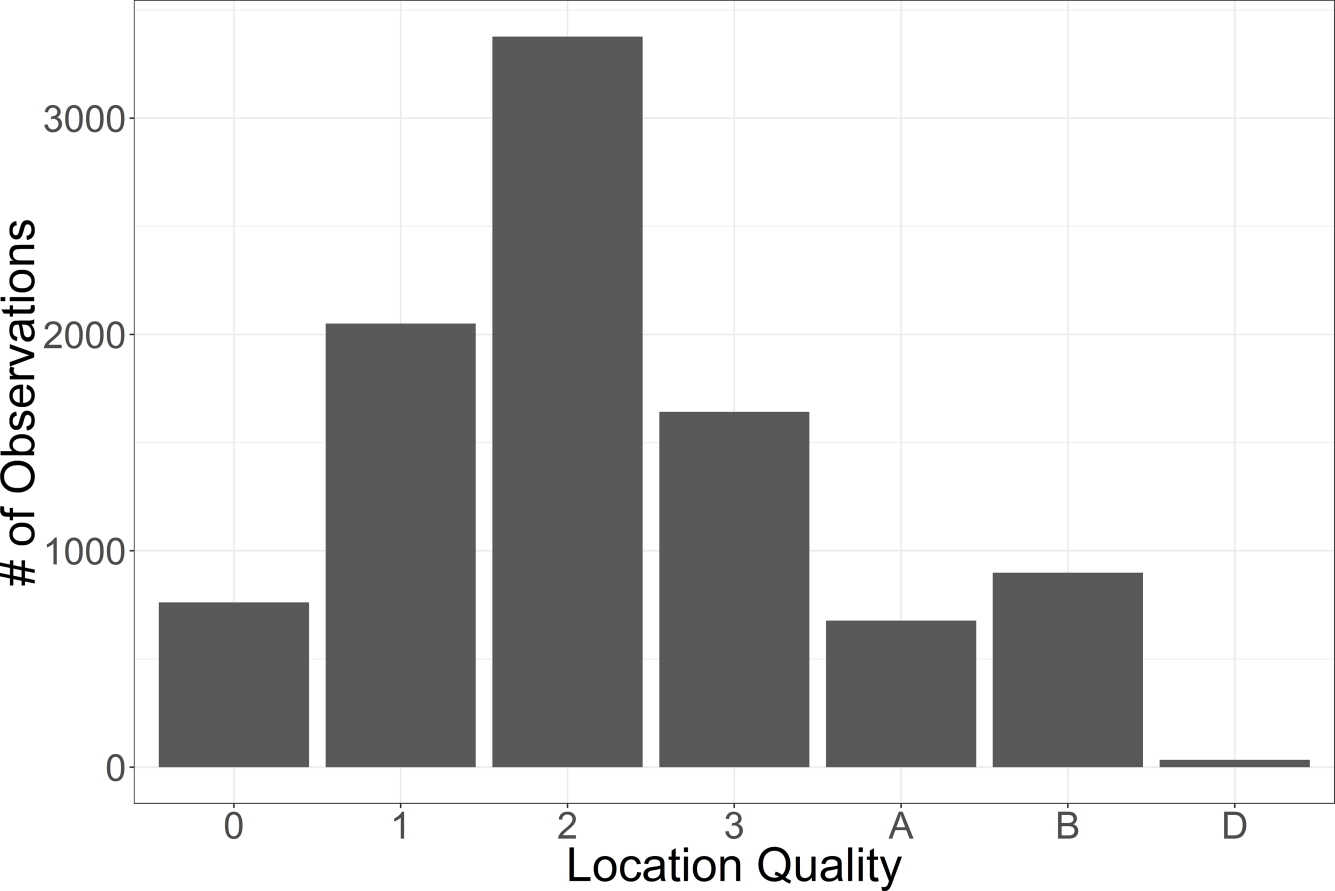
Tag location quality histogram. Histogram of SLRT location quality (best to worst from left to right with the exception of D, which is the deployment location).

Data from 34 SLRT tags were used for model development. All tags were placed on Makos (between 152 and 259 cm TL; total length) in the Southern California Bight as part of the annual juvenile shark survey conducted by the NOAA Southwest Fisheries Science Center (SWFSC) [38]. Tags were deployed between 2004–2015 primarily during the summer (June-August). All individuals had tracking data that covered at least 1 year with an average of 278 locations per individual (±86, SD). Locations were filtered to provide no more than one location estimate per day; on days where multiple records were available only the highest quality location was kept, with location qualities ranked 3, 2, 1, 0, A, B, Z. The Mako telemetry data used here had a range of location qualities but the majority were of Argos location class 1, 2, or 3 (Fig 2), with associated errors between 326–1265 meters latitude, and 742–3498 meters longitude [39]. Location classes A and B have no accuracy estimates according to the manufacturer (Wildlife Computers, Redmond, WA). Data were not interpolated; days with no location estimates were not included in the dataset. Some large gaps of weeks or months between points existed but on average the gap between two data points was 2.08 days (± 4.15, SD).

### Variable selection

The first step in designing a model requires selecting potential variables that could influence movements. In the case of Makos, size, sex, season (a set of 4 variables, one for each season, with 1 indicating when the record is in the given season and all others zero), environmental index (in this case the Multivariate ENSO Index (MEI), North Pacific Gyre Oscillation (NPGO), and the Pacific Decadal Oscillation (PDO)), moon phase (a continuous variable from 0–1, new-full), sea surface temperature, and chlorophyll-*a* concentrations (a measure of productivity) were selected as potentially important predictors affecting movement. Variables then need to be examined to ensure the scales, both spatial and temporal, match the scales of the telemetry data. For instance, latitudinal and longitudinal position estimate errors varied due to a number of factors (animal behavior, time of year, etc.) so temperature and chlorophyll-*a* concentrations were gathered from ERDDAP (Environmental Research Division's Data Access Program) with “xtracto” (R package: xtractomatic) using longitude and latitude errors based on the location class position estimate error [39], thus matching the scales of the position estimate errors with those of SST and chlorophyll-*a*. The spatial and temporal coverage of the telemetry data dictated which ERDDAP datasets could be used. Unfortunately, chlorophyll-*a* data which matched the span of the Mako tagging data only existed on a monthly average scale. Relating the movement of Makos to a monthly average of chlorophyll-*a* was considered to be of little value and was removed. SST, on the other hand, was available daily for the entire dataset from the Multi-scale Ultra-high Resolution (MUR) SST dataset. In this way, location qualities and their associated errors were used to correctly bound the scale of matching SST data, while also identifying the mismatch between our movement data and available chlorophyll-*a* data.

Once the variables are checked for data quality, we recommend that each variable be normalized so that the parameter estimates are not too small or too large in magnitude that they are subject to issues of imprecision [40]. For example, the values of length are much larger than those for SST or MEI, consequently, we normalized length using a z-score standardization:

$$Y_{i}=\frac{X_{i}-\bar{x}}{s_{X}}$$ (1)

Where *Y*_*i*_ is the standardized value, *X*_*i*_ is the measured value, $\bar{x}$ is the sample mean for that variable, and *s*_*X*_ is the sample standard deviation for that variable.

After variable normalization, a linear model can be built of the form:

$$\mu =\beta_{0}+\beta_{1}D_{1}+\beta_{2}D_{2}+\ldots +\beta_{n}D_{n}$$ (2)

where a logistic transformation of *μ* is the probability of the response variable being '1', *β*_0_ is the intercept term, *β*_1_ through *β*_*n*_ are the parameter estimates for each variable, and *D*_1_ through *D*_*n*_ represent the data for each variable, respectively.

In order to quantitatively determine which of the selected variables impact the response variable “z” (which is binomially drawn from a Bernoulli distribution where 0 indicates that the animal is east, or 1 that the animal is west of 125˚W), we ran a classification Random Forest analysis (R package: randomForest [41, 42]). Random Forest is an extension of classification and regression tree (CART) analysis, in which subsets of both the variables and the data are randomly pulled to build bifurcating classification trees, which are then internally validated by testing the performance on the remaining out-of-bag data. The importance of each variable is determined by the success of the trees including that variable. We used a total of 10,000 trees for the forest (ntree = 10000) and assessed each with an OOB sample of 1/3 of the total dataset [e.g. 43, 44]. As discussed in Strobl et al. [45] subsampling with replacement was set to false (replace = FALSE) as a precaution in order to avoid potential problems associated with variable selection when predictor variable scales are dissimilar. Since the number of data points west of the arbitrary boundary (2759) were fewer than those east (6681), the “sampsize” command was used to balance the building of the trees (sampsize was set to 1000 for each class). The significance of each predictor on the response variable was tested using “rfPermute” (R package: rfPermute (Archer 2013)) with “nrep”, the number of permutation replicates to run to construct the null distribution and calculate p-values, set to 100, and the significance level “a” set to 0.1. The calculated p-values given by rfPermute were used in preference to the basic Gini index scores produced by the “randomForrest” package to identify important predictors. If any of the predictor variables are perfect predictors, they must be removed prior to running Random Forest in order to not overfit the model. In this case, an example of a perfect predictor would be longitude, since any value above or below 125˚W would perfectly predict whether an animal was east or west of 125˚W.

# Run permuted Random Forest on Mako data

rp <- rfPermute(formula = z ~., data = Original, sampsize = c(1000,1000),

replace = FALSE, ntree = 10000, nrep = 100, a = 0.1)

For this and all subsequent example code our Mako data was stored in a data frame named “Original” with the following structure:

**head**(Original)

##     ptt sex Spring Summer Fall Winter          L z NPGO_Index PDO_Index

## 1 41676    M      0      1      0      0–0.3740933 0      0.78      0.04

## 2 41676    M      0      1      0      0–0.3740933 0      0.78      0.04

## 3 41676    M      0      1      0      0–0.3740933 0      0.78      0.04

## 4 41676    M      0      1      0      0–0.3740933 0      0.41      0.44

## 5 41676    M      0      1      0      0–0.3740933 0      0.41      0.44

## 6 41676    M      0      1      0      0–0.3740933 0      0.41      0.44

##    MEI_Index      Moon      sst      chl

## 1    0.283 0.0586144 17.45300 0.4292030

## 2    0.283 0.3222741 19.61117 0.6521956

## 3    0.283 0.7947392 19.07908 0.4757891

## 4    0.527 0.9359048 18.95387 0.3204745

## 5    0.527 0.8210133 18.47167 0.8794785

## 6    0.527 0.5869345 20.05420 0.6362990

where ptt (a unique tag number for each animal), sex, each season, and the response variable z are all saved as factors.

Following the refinement of potential predictor variables by Random Forest, the assumption of linearity was tested by running a Generalized Additive Mixed Model (GAMM) (package: gamm4 [46]). GAMM is designed to test and identify if the assumed linear relationship between response and predictor variables is appropriate, or if ‘smooths’ are needed to correctly describe a predictor variable’s relationship with the response variable [47]. By running an analysis of variance (ANOVA) on the GAMM results it is possible to identify the degrees of freedom for each predictor variable. A variable with a single degree of freedom can be treated as linear while variables with more than one degree of freedom indicate that higher order terms need to be included in the model. Setting “random” = ~(1|ptt) indicated that ptt was the random effect for the model, while specifying “family” = binomial(link = "logit") identified that the response variable is drawn from a Bernoulli distribution. Fall is set as the intercept in this model and so is not explicitly listed as one of the predictor variables.

# GAMM run on the response variable z, random effect set to individual (ptt), # and the family set to binominal with a logit link function

gamm1.0 <- gamm4(z ~ Spring + Summer + Winter + L + sex + MEI_Index,

                random = ~(1|ptt),

                data = Original,

                family = binomial(link = "logit"))

# ANOVA run on GAMM results to investigate degrees of freedom per parameter anova(gamm1.0$gam)

After establishing our predictor variables of interest with Random Forest, and testing them for linearity using GAMM, our model looked as such:

$$\mu =\beta_{0}+\beta_{1}*Spring+\beta_{2}*Summer+\beta_{3}*Winter+\beta_{4}*L+\beta_{5}*Sex+\beta_{6}*MEI$$ (3)

### Model development

The next step is the construction of the Bayesian movement model. We used Markov chain Monte Carlo (MCMC) methods to generate posterior probability distributions for model parameters. While it is possible to include prior information into Bayesian models, our approach here incorporates vague priors because often in data limited situations no prior information exists. Also, the use of vague priors in our Bayesian analysis allows for a more direct comparison to maximum likelihood model results. To deal with the nested nature of the data, i.e. multiple data points for each individual, we used a hierarchical model structured by individual tag number, which is analogous to a random effect in a generalized linear mixed model [48]. This hierarchical structure need not be based on individuals, some other possible hierarchies may be pod of whales, pack of wolves, or perhaps sample site for reef fish. The hierarchical structure of these models is versatile and can be easily adapted to fit most situations. Due to the construction of our model by individual, it’s essential that data be ordered by tag number, or similar individual identifier, in order for the hierarchical structure to be implemented correctly. First, we determine the number of data points for each individual tag (tracks) and then create a vector which specifies the first record for each tag (tracks_2), finally we use “cumsum” to identify the starting row, and ending row plus one, for each tag (cumul_tracks). This vector can then be used in the model by identifying the first location cumul_tracks[i] and last location cumul_tracks[i+1]-1 for each individual.

# Track lengths

    tracks <- table(Original$ptt)

    tracks_2 <- c(1,tracks)

    cumul_tracks <- cumsum(tracks_2)

# Number of unique tags in the dataset

    N <- length(unique(Original$ptt))

Next the model is specified as a text file within R, in our case saved with the name “model_string1.0”:

# Model specification in JAGS syntax

model_string1.0 <- "model{

    B0 ~ dnorm(0, 0.1)

    B1 ~ dnorm(0, 0.1)

    B2 ~ dnorm(0, 0.1)

    B3 ~ dnorm(0, 0.1)

    B4 ~ dnorm(0, 0.1)

    B5 ~ dnorm(0, 0.1)

    B6 ~ dnorm(0, 0.1)

    tau ~ dgamma(0.1, 0.01)

    s <- 1/sqrt(tau)

      for(j in 1:N) {u[j] ~ dnorm(0, tau)

        for(i in cumul_tracks[j]:(cumul_tracks[j+1]-1)) {

          logit(mu[i]) <- B0 + (B1 * Spring[i]) + (B2 * Summer[i]) +

          (B3 * Winter[i]) + (B4 * L[i]) + (B5 * Sex[i]) +

          (B6 * MEI[i]) + u[j]

          z[i] ~ dbern(mu[i])

        }

      }

  }"

where the B terms are the parameters for each variable in the model. Because there were no previous data to populate informed priors, the model is designed to include vague priors from a normal distribution with a mean of 0 and a variance of 10 (JAGS uses precision which is 1/variance in dnorm so a variance of 10 in JAGS is defined as 0.1). Tau is drawn from a gamma distribution with a mean of 10 (mean = 0.1/0.01) and a variance of 1000 (variance = 0.1/(0.01)^2^) and used to define the model process error “s”, which is error that is not accounted for by individual variation. Individual variability is assigned to “u” and allowed to vary for each loop of “j”. Since the response variable is binomial, the model is set with a logit link function and the response variable “z” is drawn from a Bernoulli distribution. We then run the model using rjags (R package: rjags [49, 50]) and save the result to the object “model1.0”.

# Run JAGS model and specify initial parameter values

model1.0 <- jags.model(textConnection(model_string1.0),

                            n.chains = 4,

                            n.adapt = 100000,

                            data = list(Spring = Original$Spring,

                                    Summer = Original$Summer,

                                    Winter = Original$Winter,

                                    L = Original$L,

                                    Sex = Original$sex,

                                    MEI = Original$MEI_Index,

                                    z = Original$z,

                                    N = N,

                                    cumul_tracks = as.vector(cumul_tracks)),

                            inits = function ()

                            {

                              list('B0' = runif(1, 0, 1),

                                   'B1' = runif(1, 0, 1),

                                   'B2' = runif(1, 0, 1),

                                   'B3' = runif(1, 0, 1),

                                   'B4' = runif(1, 0, 1),

                                   'B5' = runif(1, 0, 1),

                                   'B6' = runif(1, 0, 1))

                            },)

    update(model1.0, 10000)

Within the rjags command we specify our model (as saved in the text file above), the number of parallel chains for the model with “n.chains”, the number of iterations for adaptation with “n.adapt”, the data (as a list drawn from our data frame “Original” and the previously created cumul_tracks object), and a list of initial starting values for each parameter, in this case a single random value drawn from a uniform distribution between zero and one. The uniform distribution is used here so that any value between zero and one is just as likely to be selected as any other, unlike a normal distribution where values will typically come from near the mean. The starting point is not particularly important as the chains will eventually converge to a posterior distribution given sufficient steps. However, using starting points that are close to the resulting posterior distribution would make the computation more efficient [51]. The function “update” is then used to update the Markov chain associated with the model, with “n.iter” set to 10,000 indicating the number of iterations of each Markov chain to run.

Posterior samples are then coerced into a single mcmc.list object (draws1.0) using “coda.samples” [49]:

# Runs coda and identifies parameters to monitor

draws1.0 <- coda.samples(model = model1.0,

                    1n.iter = 500000,

                    1variable.names = c("B0", "B1", "B2",

                                        "B3", "B4", "B5",

                                        "B6", "s", "u"),

                    thin = 10)

where model1.0 specifies the JAGS model, 500,000 indicates the number of iterations to monitor, “variable.names” indicates the variables of interest that we want to be tracked and reported on (in this case all of the B parameters, model process error (s), and individual variability (u)), and 10 is the thinning interval set so that only every 10^th^ value out of all MCMC iterations will be saved [51]. Results from coda.samples are stored in the “draws1.0” object.

An important step in our Bayesian model development is to test the assumption that our priors are truly vague and not unintentionally influencing the model. To test this we specified a priors-only (data-free) model “model_string0.0”:

# Priors-only model specification in JAGS syntax

model_string0.0 <- "model{

    B0 ~ dnorm(0, 0.1)

    B1 ~ dnorm(0, 0.1)

    B2 ~ dnorm(0, 0.1)

    B3 ~ dnorm(0, 0.1)

    B4 ~ dnorm(0, 0.1)

    B5 ~ dnorm(0, 0.1)

    B6 ~ dnorm(0, 0.1)

    tau ~ dgamma(0.1, 0.01)

    s <- 1/sqrt(tau)

      for(j in 1:N) {u[j] ~ dnorm(0, tau)

        for(i in cumul_tracks[j]:(cumul_tracks[j+1]-1)) {

          logit(mu[i]) <- B0 + (B1) + (B2) + (B3) + (B4) + (B5) + (B6) + u[j]

          z[i] ~ dbern(mu[i])

        }

      }

  }"

which we then run as before using rjags but with only the response variable “z” in the data list. When plotted on the same scale as the posterior distribution of the data-full model, the posterior of the priors-only model should be nearly undetectable if priors are truly vague. If instead plots indicate that the priors-only posteriors are detectible, then the priors are still having an impact on the model results and need to be expanded.

Models were also constructed to estimate parameters using maximum likelihood estimation (MLE). Despite the philosophical differences between Bayesian and MLE approaches, with vague priors, the parameter estimates from both approaches should be similar [52, 53]. We implemented GLMMs with individual tag number as a random effect to account for the nested nature of repeated sampling within individuals using:

1) glmer using the Laplace approximation in the R package lme4 [31]

glmer1.0 <- glmer(z ~ Spring + Summer + Winter + L + sex + MEI + (1|ptt),

                family = binomial(link = "logit"), data = Original)

and

2) Penalized quasi-likelihood PQL in the R package MASS [54]

glmm_pql <- glmmPQL(z ~ Spring + Summer + Winter + L + sex + MEI,

                    random = ~ 1|ptt, family = binomial, data = Original)

where z is the binomial response variable followed by each variable of interest. Both methods, glmer and glmmPQL have slightly different syntax for indicating random effects and the family of the distribution but the concept remains the same. Penalized quasi-likelihood is the simplest and most widely used GLMM method despite known issues of biased parameter estimates if the standard deviations of the random effects are large, especially with binary data [30–32]. Our use of PQL is meant as a comparison to perhaps more appropriate methods given the nature of our data, an important point of discussion given PQLs ubiquity in the literature. Glmer is a more suitable GLMM approach for the data used here.

### Model performance with data paucity

Since one of the primary reasons that many previous telemetry studies lack population level inferences is the absence of large datasets, we decided to test both the Bayesian and MLE approaches on pared down subsets of the full dataset of 9440 locations. We randomly selected 25 replicates each of 75, 50, 25, and 10 percent of the full dataset (7080, 4720, 2360, 944 data points respectively) resulting in 100 new, “data poor” datasets. Data subsetting was done without concern for individuals, i.e. individuals were not selectively removed to make smaller datasets, instead points were randomly removed across the whole dataset. Each of these data poor datasets were analyzed using the Bayesian and the two MLE approaches. Mean parameter estimates from each level of data paucity were compared to the congruous parameter estimates calculated using the full dataset to evaluate accuracy. The variance in parameter estimates for each level of data paucity were also examined to test the precision of these models given the smaller sample sizes from a larger population.

### Test of model performance with simulated data

While the pared down datasets function as a useful example of model performance with real world data, to truly test model performance, simulated data were created with known parameter values and then run through the model to see if the parameter values could be recovered. Code for creating a simulated dataset can be found in the Appendix. We created 10,000 unique simulated datasets each with a comparable number of individuals (n = 34) and data points per individual (~278) as the Mako dataset. Individual variation was accounted for by including an error term in the simulation code which was comparable to the amount of individual variation in the real Mako dataset. Testing a model by simulating data and then recovering the parameter estimates used to create it is one of the best ways to test model performance, however a simulation test is not a necessary step in creating a movement model and hence its discussion here is brief and only focused on model performance.

### Spatial and temporal autocorrelation

We focus here on movement models that treat data points from an individual fish as independent from one another. We recognize that in many cases spatial and temporal autocorrelation can have a major impact on analyses and inferences from tagging studies [55–58]. Often, where an animal is at time x effects where it will be at time x+1. Much time and effort have been spent grappling with this issue and no single method for dealing with it has yet been accepted in the scientific literature. We built a version of our Bayesian model that accounts for this autocorrelation (S2 Appendix). After running the autocorrelated model, we compared the results with the non-autocorrelated model described in this paper and found that the results were comparable (S2 Appendix). Due to the increased simplicity of the non-autocorrelated model, easier to interpret results, and reduced running time, we opted to focus on the simpler model. We suggest that anyone interested in applying these models to telemetry data should consider if the autocorrelated model is more appropriate for their dataset. If unsure, both models should be run and the results compared.

## Results

**Fig 3:**
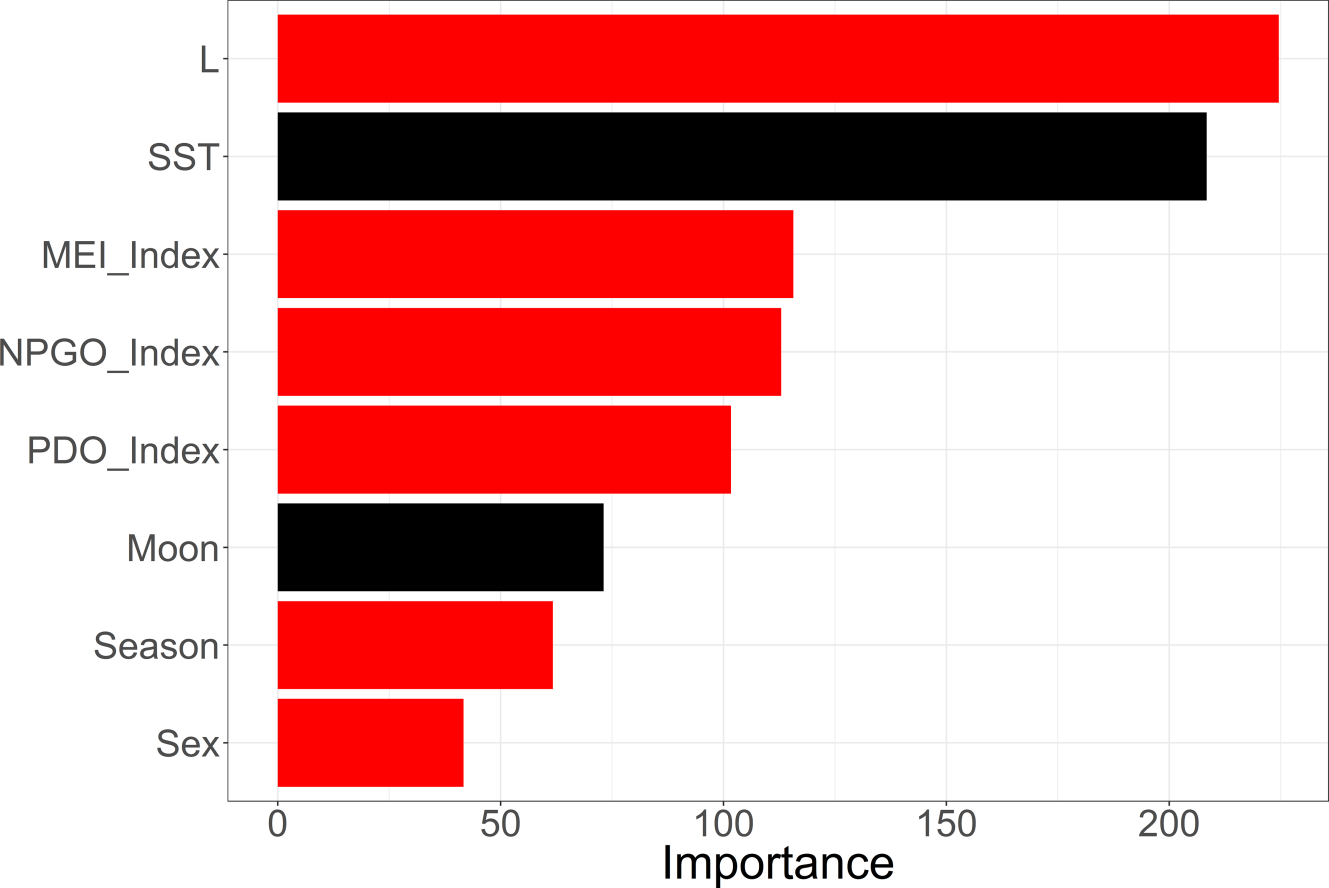
Gini index of relative parameter importance. Gini index of importance scores for all of the potential parameters in the initial permuted Random Forest run (including three different environmental indices). Red indicates a parameter that significantly partitioned the data. Tested variables: Length, sea surface temperature, Multivariate ENSO Index (MEI), North Pacific Gyre Oscillation (NPGO), and the Pacific Decadal Oscillation (PDO), moon phase, season, and sex.

**Fig 4:**
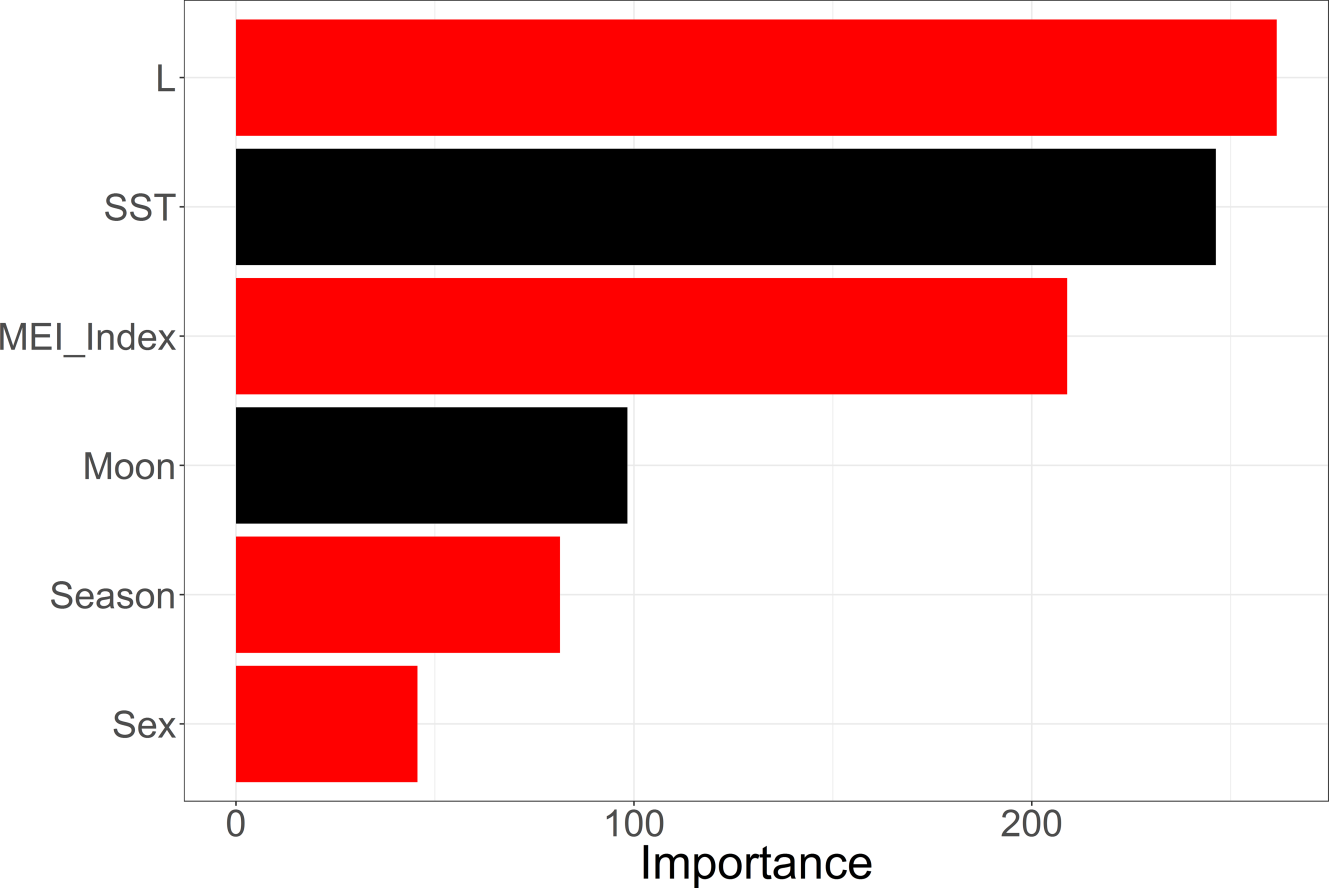
Gini index of relative parameter importance. Gini index of importance scores for all of the potential parameters in the ultimate permuted Random Forest run (including the single most influential environmental index, MEI). Red indicates a parameter that significantly partitioned the data.

Using the full set of Mako data, results from the Random Forest analysis indicated that our OOB classification error rate was low, 8.76%, with misclassification of zeros (points east of 125˚W) slightly higher than misclassification of ones (confusion matrix class error 0.09 and 0.07, respectively). The Gini importance measure indicated that length was the most influential model parameter, followed by the environmental indices, season, and sex. Moon phase, and sea surface temperature were found to be non-significant classification variables (Fig 3). With the ability of Random Forests to handle correlated variables we tested three different environmental indices: NPGO (North Pacific Gyre Oscillation), MEI (Multivariate ENSO Index), and PDO (Pacific Decadal Oscillation), simultaneously in order to determine which was best suited to use as a predictor in our later modeling approaches. The multivariate ENSO Index was identified as the most influential index and subsequently run in a separate Random Forest without the other two indices. Dropping the other indices had no effect on which parameters were identified as important by the Gini index (Fig 4) giving us confidence in our selected model parameters.

Results from the GAMM indicated that all tested parameters had one degree of freedom and hence could be treated as linear, no higher order terms were needed (Table 1).

**Table 1:** Results from GAMM indicating degrees of freedom for each parameter identified as influential by random forest.

	DF	Chi.sq	p-value
**Spring**	1	88.31	<0.05
**Summer**	1	176.21	<0.05
**Winter**	1	0.19	0.66
**L**	1	6.04	0.01
**Sex**	1	0.02	0.89
**MEI_Index**	1	112.70	<0.05

**Fig 5:**
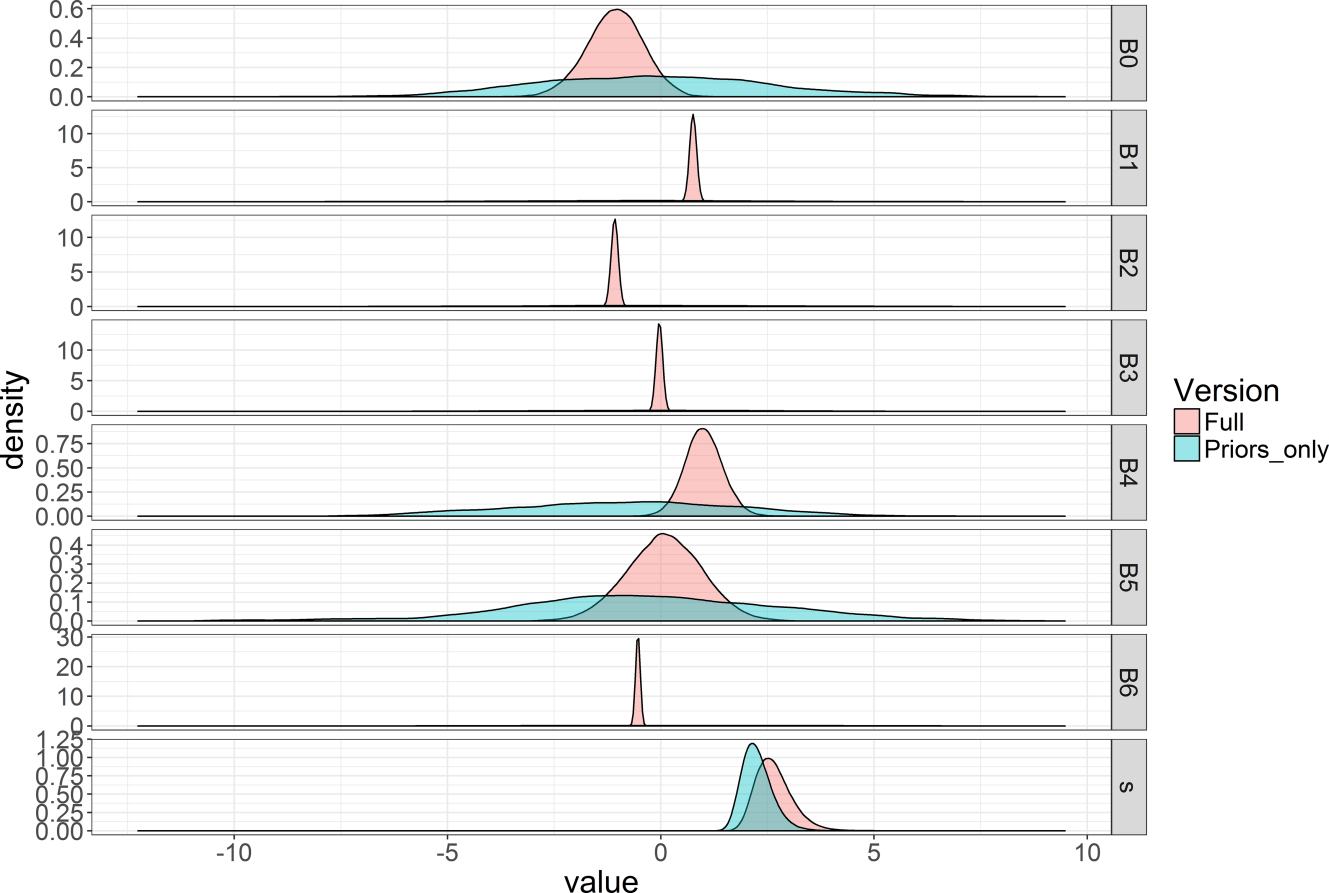
Bayesian posterior distribution plots with identical priors. Bayesian posterior distributions for all of the parameters included in the model with identical priors. All priors were defined as normal distributions with a mean of 0 and a variance of 10. Red indicates the model with both data and priors and blue indicates the prior-only model.

For our Bayesian approach a test of the influence of model priors was done by comparing the full Bayesian data model with our priors-only model, where all beta parameter priors were set with a mean of 0 and a variance of 10. The priors for B0, B4, and B5 appeared more informative then intended (Fig 5). To rectify this we designated broader distributions by increasing the variance for the priors of these parameters.

**Fig 6:**
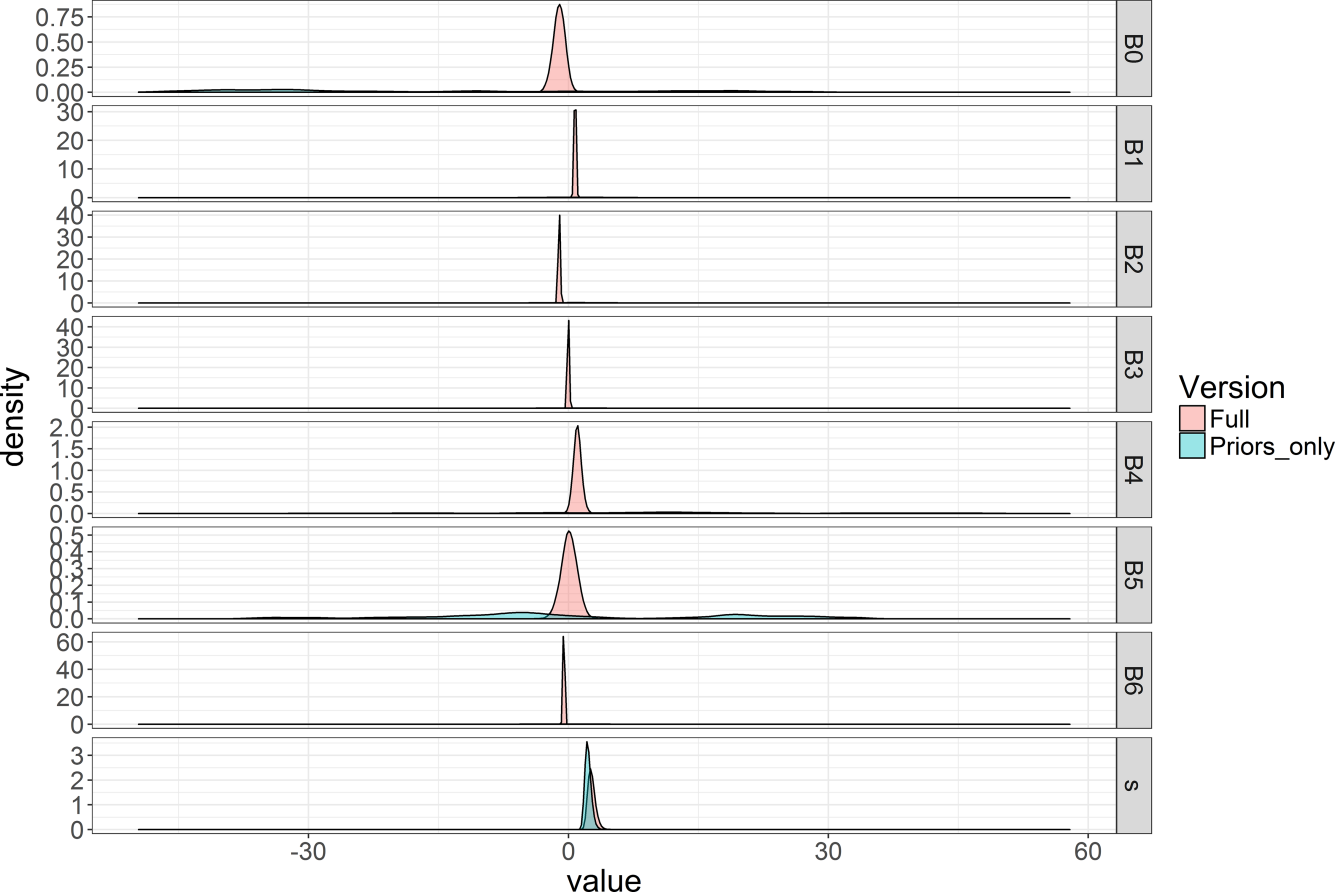
Bayesian posterior distribution plots with expanded, flat priors. Bayesian posterior distributions for all of the parameters included in the ultimate model with vague priors. All priors were defined as normal distributions with a mean of 0 and a variance large enough to not influence the posterior distribution for that parameter. Red indicates the model with both data and priors and blue indicates the prior-only model.

A rerunning of the priors-only model indicated that the expanded variance of B0, B4, and B5 achieved the desired effect, alleviating concerns of unintentionally informative priors (Fig 6).

**Fig 7:**
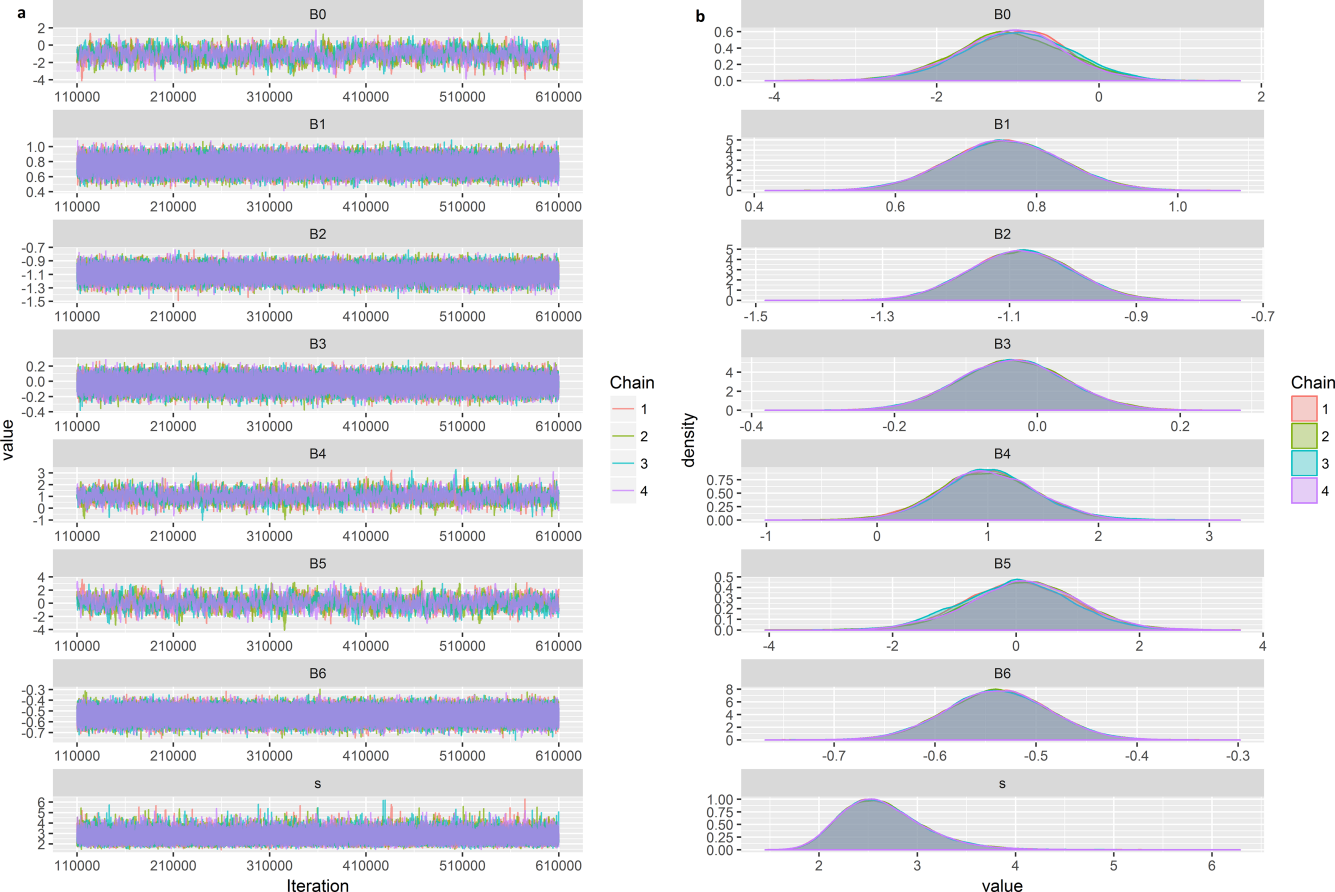
Trace plots and Bayesian posterior plots for the final model. Trace plots indicating good mixing of the four model chains (a) and posterior distribution plots indicating parameter estimates produced by the model (b).

With revised vague priors the Bayesian model showed good indications of convergence, with “grassy” trace plots indicating good mixing among chains (Fig 7A), normal distributions for each parameter’s posterior (Fig 7B), and Gelman scores between 1 and 1.01 for all parameters. Posteriors indicated that winter and fall were statistically similar (fall being the intercept and winter having a posterior distribution which centered near zero with a mean of -0.034, CI = -0.183, 0.115). Spring and summer both differed from fall with posterior 95% credibility intervals which did not encompass zero (spring: mean = 0.756, CI = 0.598, 0.914; summer: mean = -1.082, CI = -1.242, -0.924). Length also had a 95% credibility interval outside of zero with a mean of 1.007, CI = 0.120, 2.026. The MEI index posterior also indicated a distribution which did not encompass zero, with a mean of -0.538, CI = -0.638, -0.439. The effect of sex was minimal with mean and 95% credibility interval centered on zero (mean = 0.086, CI = -1.812, 1.919) (Fig 7B).

**Fig 8:**
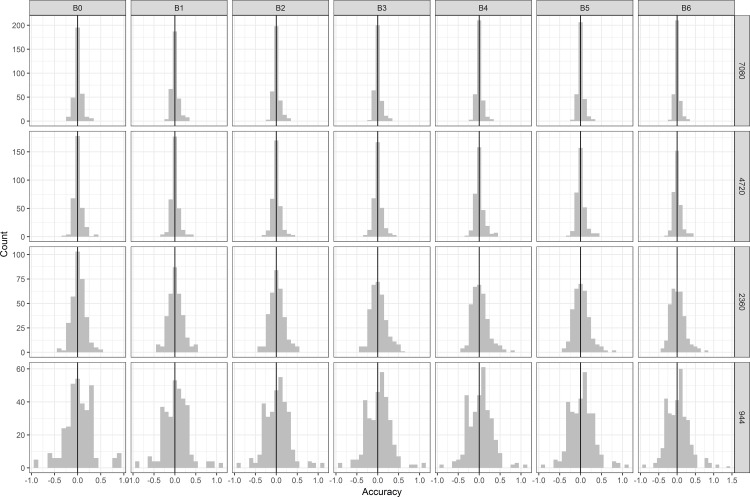
Comparison of Bayesian parameter estimates given varying amount of data. Accuracies of parameter estimates with different amounts of data. Accuracy was determined by subtracting the parameter value estimated using the full dataset from the estimate using reduced datasets. Columns are arranged by parameter and rows indicate that size of the dataset used.

With vague priors and the same parameters, results from the Bayesian and Maximum Likelihood approaches were comparable (Table 2). This remained true even as the models were provided with reduced datasets. To avoid redundancy, we will focus on the results from only the Bayesian models in our data paucity tests. As would be expected, the accuracy of mean parameter estimates decreased as models were provided with less and less data. When compared to estimates from the full dataset, minimum and maximum mean estimates across all parameters expanded from -0.24 to 0.33 when given 75% of the data up to -0.88 to 1.39 when given only 10% of the original data (Fig 8). Precision (as measured by 95% credibility interval width) also decreased with data paucity, with the median credibility interval width for most parameters expanding by ~0.56 when only 10% of the data were used compared to when all the data were used. Despite these decreases in accuracy and precision, inferences (point estimates) from the models did not change, although confidence in those inferences was reduced.

**Table 2:** Parameter means and the random effect standard deviation from all three tested models, JAGs (Bayesian), glmer, and glmPQL.

model	data_size	B0.mean	B1.mean	B2.mean	B3.mean	B4.mean	B5.mean	B6.mean	RandEff_SD
JAGS	9440	-1.07184	0.75605	-1.08214	-0.03431	1.007088	0.08584	-0.53784	2.655815
glmer	9440	-1.42083	0.755772	-1.08019	-0.03308	0.996651	0.118426	-0.53526	2.401489
glmPQL	9440	-1.33962	0.751796	-1.07625	-0.03297	0.920856	0.139784	-0.53176	2.234407

**Fig 9:**
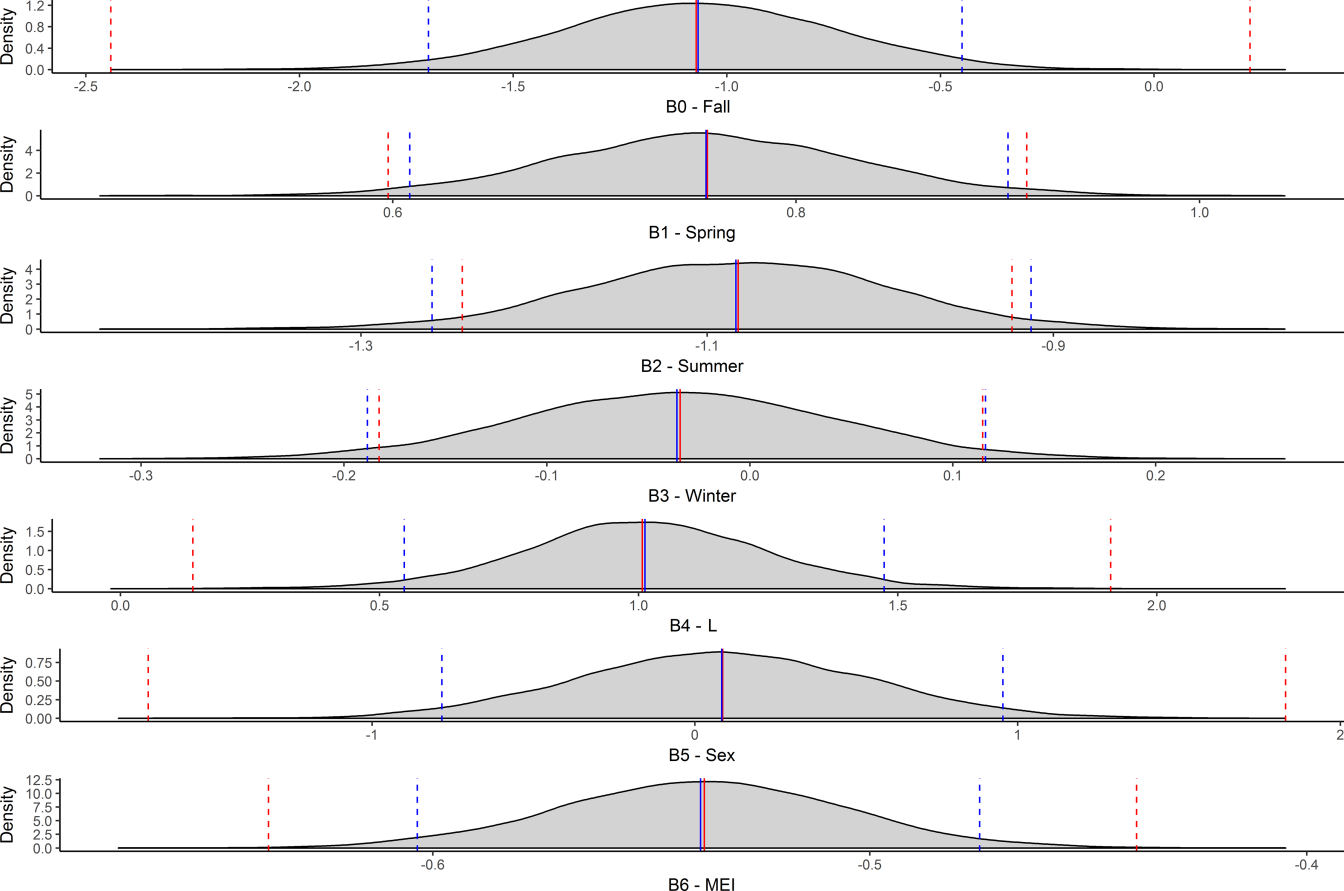
Comparison of GLMM parameter estimates from different sized simulated datasets. Density plots for parameter estimates across 10,000 simulated datasets. Solid red lines indicate true parameter values used to create simulated data, solid blue lines represent the median estimate produced across the 10,000 model runs. Dashed blue lines indicate the 95% credibility intervals for each parameter, for comparison dashed red lines indicate the 95% credibility intervals for each parameter given the real Mako data.

Results from the simulation analyses indicated that the Bayesian approach performed well and was able to recapture the true parameter values used to create the 10,000 simulated datasets (Fig 9). Mean parameter estimates were nearly identical to the simulated values used to create the data, confidence intervals were also similar to the full model’s 95% credibility intervals indicating that estimates were reliably recovered across all simulated datasets. As with the subset datasets, adding more individual variability to the simulated datasets increased the widths of the confidence intervals but had little to no effect on the point estimates, again indicating that inferences would remain stable but confidence in those inferences would decline.

## Discussion

The interpretation of model results here is brief since as stated previously, the question investigated in this analysis was not selected for its ecological relevance to Mako movement. Instead, we will focus on discussing the models themselves, how they performed at different levels of data paucity, and how to interpreting their results, rather than the specific conclusions related to Mako movement.

Firstly, we note that the effect of expanding prior variance in our case was negligible, indicating that with the tested sample size our data was overriding the influence of the priors even before they were broadened. This has been seen in other Bayesian studies with even smaller sample sizes [59]. Despite the limited effect of expanding our priors in this analysis, the exercise of comparing the posteriors of the priors-only model to the full model is useful in gaining a more complete understanding of one’s data and how specified priors may be affecting it.

The influence of covariates on horizontal movement can be interpreted by assessing the values of Bayesian estimates (mean, medians, modes, etc. of posterior distributions) and whether the 95% credibility intervals include zero [60]. Covariates that did not include zero within the credibility intervals were considered to significantly (with 95% probability) contribute to movement east or west of 125˚W. Positive estimates were interpreted as covariates that are positively related to movement west of 125˚W, while negative estimates are related to movements east of 125˚W. Since our question was not ecologically relevant to Mako movement we will not attempt to over-interpret our model results, however, as an example of how our results could be interpreted we will indicate how model results for something like length could be interpreted. With a positively shifted posterior whose 95% CI does not include zero we could indicate that animal length affected Mako movements, i.e. larger length values are positively correlated with z values of 1, in other words, large Makos were more likely to travel west of 125˚W (Fig 7).

When subset datasets were analyzed with each model, credibility intervals expanded to different degrees around each variable. However, the point estimates themselves did not change, variables with significant, positive estimates continued to have positive estimates as the dataset was reduced. Similarly, those variables with negative estimates continued to be negative as the dataset became increasingly sparse. This result suggests that researchers with data limited telemetry datasets should attempt to quantitatively pursue population level inference. It is important to note that the increased credibility interval with data limitations may cause a Bayesian posterior to broaden enough to include zero. For this reason, it is important to consider whether the amount of data is particularly sparse before determining that a predictor variable is not significantly influencing a model. One of the strengths of the Bayesian approach is how scarcity in data results in larger uncertainty in parameter estimates. A posterior distribution of the coefficient for a predictor variable may include zero because there were not enough data to elucidate the strengths of the predictor. The entire uncertainty about this parameter, given the data and model, is provided in the posterior distribution where one can compute the exact probability of this coefficient being positive or negative.

As computing power and the accessibility of advanced statistical approaches via programs like R increase, methods for analyzing complex telemetry datasets are rapidly expanding. Hierarchical, mixed-effects, and state-space models, using Bayesian or maximum likelihood analytical approaches, are part of the developing toolbox available to ecologists [61, 62]. Each of these approaches holds its own benefits and drawbacks but when used properly can allow telemetry researchers to quantitatively address questions of animal movement at the population level. Here we have presented results from hierarchical and mixed-effects models with both Bayesian and MLE approaches and specifically examined effects of data paucity on population-level inference. Our results indicate that given a properly framed question that takes account of the scale of both the data and the predictor variables of interest, both the Bayesian approach with vague priors and the MLE approach produce similar inferences, even as the datasets were reduced in size. However, the Bayesian approach provides several advantages for both current and future studies.

A major advantage to the Bayesian approach is that all results are exact; there are no asymptotic assumptions involved in the estimates as with MLEs, which may be of questionable value in data-limited situations which are typical of ecological studies [48]. Additionally, Bayesian credibility intervals have a more natural interpretation than classical confidence intervals. For example, the inference from the 95% credible interval for the coefficient for Length is that the true value of this parameter lies within (0.120, and 2.026), with probability 0.95, given the data and the model. Within classical or frequentist statistics, such a statement is invalid because the parameters are assumed to be fixed [63]. While not restricted to Bayesian analysis alone, we think it is important to utilize a hierarchical structure (when warranted) no matter what modeling approach is taken. As presented here, a hierarchical structure allows for a probabilistic link from parameters at the individual level to parameters at the population level [64, 65]. This structure affords a population level understanding of particular behaviors by borrowing strength across the individual datasets [64, 66, 67].

The use of priors can also be an advantage in Bayesian analysis. In the work presented here we avoided the use of informative priors for two reasons: 1) to allow a more direct comparison of results between Bayesian and MLE approaches, and 2) because in data-limited situations, prior information may not be available and so it is important that vague priors still produce credible results. This speaks to the use of priors in the current analysis. The ability to utilize priors, however, can also be beneficial to future studies. For example, assuming an interesting and valid question, posterior estimates from a recent study can easily be adapted into priors in a future study. As is often the case with highly migratory pelagic species like the Mako example discussed here, tracking studies are expensive and time consuming, and analyses are often conducted on relatively small datasets which limits confidence in study inferences. It is unlikely, however, that such a study will be the last ever conducted on that species, e.g. research on Mako movement in the Pacific is likely to continue long into the future. With the ability of the Bayesian approach to integrate prior knowledge into current analysis, it would be relatively easy to take posterior distributions from one study and use them as prior distributions in a future study. This would allow movement studies to obtain more precise parameter estimates by leveraging information from the posterior distributions of previous investigations without having to source all the previous studies raw data.

The analysis of animal movements using telemetry data is filled with subjective choices, from the filtering of location estimates and the selection of predictor variables, to the analytical method itself. Careful thought must go in to each step and no one approach will be suited for all datasets or all questions. Here we have presented a step by step approach to the analysis of telemetry data with a binominal question, we have discussed results from MLE and Bayesian approaches, and investigated the effect of data paucity on model inferences. We have indicated the benefits of a Bayesian approach, especially in data-limited situations, and also acknowledged the performance of MLE methods. With this framework, we hope to spur other researchers to apply these kinds of population level quantitative approaches to their own data, even in data-limited situations.

## Supporting information

S1 Appendix R code for creating simulated datasets. (DOCX)

S2 Appendix Discussion of autocorrelation and its effect on the modeling of the presented mako movement data, including a figure comparing results from the autocorrelated and non-autocorrelated models. (DOCX)
