# Supplementary material for: An analytical approach to sparse telemetry data
Source: PLoS One. 2017 Nov 28;12(11):e0188660. doi: 10.1371/journal.pone.0188660 (PMC5705164; doi:10.1371/journal.pone.0188660)
Supplement: S1 Appendix — (DOCX) [file pone.0188660.s001.docx]

Quantitative approach for analyzing telemetry data in data limited situations: S1 Appendix

Michael Kinney

July 27, 2016

###### Create simulated data

library(boot)
library(lme4)

## Loading required package: Matrix

library(plyr)

##### Data Function #####
# Default beta values set to real mako data model results
data.fn <- function(ks=points, B0 = -1.07184235363113, B1 = 0.756049892254036, B2 = -1.08214012181972, B3=-0.034312857681891, B4=1.00708760231013, B5=0.0858399743631905, B6=-0.537840877614442, SD.m=0.98) {

 # Season created with each season given equal likelyhood
 seasons <- t(as.data.frame(rmultinom(n = ks, size = 1, prob = c(0.25, 0.25, 0.25, 0.25))))
 # MEI drawn randomly
 MEI <- rnorm(n=ks, mean = 0.5617759, sd=0.8877218)

 # Generate fork length then r transform the data
 L.raw <- rep(rnorm(1, mean = 123.0, sd = 23),times=ks)
 L <- (L.raw-123)/23

 Sex <- rep(rbinom(n = 1, size = 1, prob = 0.5), times=ks)

 # Individual error
 u <- rep(rnorm(1, mean=0, sd=SD.m), times=ks)

 # Signal: build up systematic part
 exp.p <- B0 +
 (B1 * seasons[,2]) +
 (B2 * seasons[,3]) +
 (B3 * seasons[,4]) +
 (B4 * L) +
 (B5 * Sex) +
 (B6 * MEI) +
 u

 z <- rbinom(n=ks, size = 1, p=inv.logit(exp.p))

 return(cbind(z, ptt=i, Fall=seasons[,1], Spring=seasons[,2], Summer=seasons[,3], Winter=seasons[,4], L, Sex, MEI_Index=MEI, exp.p))
}

#### End of Function ####

 # Make an empty data frame to store sim data
 Original <- data.frame(NULL)

 # Set to run 34 times, each representing an individual, ks set to onerandom draw with a mean of 278
 for(i in 1:34){
 ks <- rpois(1, 278)
 data <- data.fn(ks=ks)

 Original <- rbind(Original, data)

 }
