# Supplementary material for: An analytical approach to sparse telemetry data
Source: PLoS One. 2017 Nov 28;12(11):e0188660. doi: 10.1371/journal.pone.0188660 (PMC5705164; doi:10.1371/journal.pone.0188660)
Supplement: S2 Appendix — (DOCX) [file pone.0188660.s002.docx]

Quantitative approach for analyzing telemetry data in data limited situations: S2 Appendix

Michael Kinney

The model presented in the main paper focuses on a movement model that assumes that an animal’s location is determined by a combination of environmental and demographic parameters. In many real systems, an animal’s current location can be confounded by spatial-temporal autocorrelation, in which its current location is influenced by its previous locations. In this case, a model has to be designed to account for this lack of independence in locational data. For such cases, we designed a model that is analogous to the model discussed in the body of this paper, but accounting for spatial-temporal autocorrelation. In the below model, the demographic and environmental parameters are incorporated similarly to the primary model, however, the mu[i-1] term is included to account for the previous location at time t-1.

###### Autocorrelated Bayes Model

model_string1.1 <- "model{
 B0 ~ dnorm(0, 0.000001)
 B1 ~ dnorm(0, 0.1)
 B2 ~ dnorm(0, 0.1)
 B3 ~ dnorm(0, 0.1)
 B4 ~ dnorm(0, 0.0001)
 B5 ~ dnorm(0, 0.000001)
 B6 ~ dnorm(0, 0.1)
 tau ~ dgamma(0.1, 0.01)
 s <- 1/sqrt(tau)
 for(j in 1:N){
 u[j] ~ dnorm(0, tau)
 #Set the first point to a zero then calculate mu for the first point
 mu[cumul_tracks[j]] <- B0 +
 (B1 * Spring[cumul_tracks[j]]) +
 (B2 * Summer[cumul_tracks[j]]) +
 (B3 * Winter[cumul_tracks[j]]) +
 (B4 * L[cumul_tracks[j]]) +
 (B5 * Sex[cumul_tracks[j]]) +
 (B6 * MEI[cumul_tracks[j]]) +
 u[j]

 for(i in (cumul_tracks[j]+1):(cumul_tracks[j+1]-1)) {
 mod.1[i] <- B0 +
 (B1 * Spring[i]) +
 (B2 * Summer[i]) +
 (B3 * Winter[i]) +
 (B4 * L[i]) +
 (B5 * Sex[i]) +
 (B6 * MEI[i]) +
 u[j]

 cov[i] ~ dnorm(mod.1[i], s)
 logit(mu[i]) <- mu[i-1] + cov[i]

 z[i] ~ dbern(mu[i])
 }
 }
 }"

In the case of these data, the autocorrelated model provides qualitatively similar parameter estimates to the non-autocorrelated model without changing population level inferences. This indicates that autocorrelation is not a major issue for these data.

###### Autocorrelated vs non-autocorrelated parameter estimates


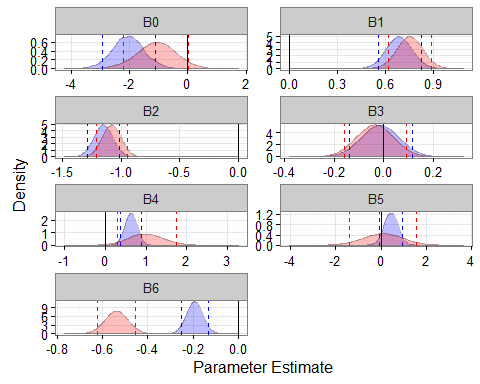

 This figure demonstrates that the parameter estimates for the non-autocorrelated model (in red) and the autocorrelated model (in blue) provide qualitatively similar results. All parameters 90% credibility intervals are shifted in the same direction across the two models. For all but B6(MEI index), posterior distributions overlap across the two models, and in the case of B6 the negatively shifted peaks still provide the same model inference.
